# Cost-utility analysis of stratified isoniazid dosing by NAT2 genotype compared with isoniazid standard regimen for new patients with pulmonary tuberculosis in Thailand

**DOI:** 10.3389/fpubh.2026.1772312

**Published:** 2026-03-27

**Authors:** Mantana Kantared, Unchalee Permsuwan

**Affiliations:** 1 Faculty of Pharmacy, Chiang Mai University, Chiang Mai, Thailand; 2 Department of Pharmaceutical Care, Faculty of Pharmacy, Chiang Mai University, Chiang Mai, Thailand

**Keywords:** cost-effectiveness, CUA, isoniazid, NAT2 genotype, pulmonary tuberculosis

## Abstract

**Background:**

Isoniazid is metabolized by the N-acetyltransferase 2 (NAT2) enzyme. Individuals classified as slow acetylators are increased risk of developing hepatotoxicity. This study aims to evaluate the clinical and economic impact of incorporating NAT2 genotype-guided dosing of isoniazid to prevent anti-tuberculosis drug-induced hepatitis in the Thai healthcare system.

**Methods:**

A decision tree and Markov model were developed to assess the costs, clinical outcomes, and quality-adjusted life years (QALYs) of NAT2 genotype-guided isoniazid dosing compared to the standard regimen in newly diagnosed pulmonary tuberculosis patients in Thailand. The primary outcomes were costs, QALYs, and incremental cost-effectiveness ratios (ICERs).

**Results:**

The analysis revealed that the discounted costs for the NAT2 genotype-stratified isoniazid dosing group were 28,538 THB (USD 870), compared to 18,727 THB (USD 570) for the standard regimen. The corresponding discounted QALYs were 9.92 years and 7.66 years, respectively. The ICER was 4,333 THB (USD 132) per QALY gained. One-way sensitivity analysis showed that the intervention was cost-effective across all input parameters. Probabilistic sensitivity analysis indicated that 99.98% of simulations were below the threshold for cost-effectiveness.

**Conclusion:**

Stratified isoniazid dosing by NAT2 genotype would be cost-effective in treatment of pulmonary tuberculosis in Thailand.

## Introduction

Tuberculosis (TB) continues to pose a significant public health burden in Thailand, with treatment success rates for new and relapsed cases consistently falling short of the World Health Organization (WHO)‘s global target of 90%. Between 2021 and 2023, Thailand reported treatment success rates of 81.7, 70.8, and 76.8%, respectively (1–3). Key contributors to these suboptimal outcomes include mortality during treatment (9.3% in 2021, 9.8% in 2022, and 8.7% in 2023) and loss to follow-up or treatment default (4.9, 5.6, and 4.4% for the same years) (1–3).

Despite ongoing efforts under national TB control programs, 78,955 new TB cases were registered in 2023. Among these, drug-induced hepatotoxicity (DIH) was reported in 6.8% of patients, which correlated with a reduced treatment success rate of 43.2% and a significantly increased mortality rate of 18.2% (4). In response, Thailand has implemented its second National Strategic Plan for Tuberculosis Control (2023–2027), targeting a reduction in TB incidence to 89 cases per 100,000 population and aiming to achieve a treatment success rate of ≥90% by 2027 (1).

Current treatment guidelines from the WHO and Thailand continue to recommend first-line anti-tuberculosis agents, including isoniazid, rifampicin, ethambutol, and pyrazinamide. However, DIH remains a substantial clinical challenge, necessitating treatment regimen modifications, prolonged therapy, and increased healthcare expenditures. The mechanisms underlying hepatotoxicity are varied: rifampicin impairs bilirubin clearance, leading to hyperbilirubinemia, and induces hepatic enzymes that accelerate isoniazid metabolism. Pyrazinamide-induced hepatotoxicity remains incompletely understood; however, it appears to be dose-dependent, especially at doses exceeding 30 mg/kg/day (5). Isoniazid is primarily metabolized by arylamine N-acetyltransferase 2 (NAT2), and its plasma concentration is strongly influenced by the individual’s NAT2 genotype. Patients with the slow acetylator phenotype frequently demonstrate supratherapeutic isoniazid concentrations (6). A systematic review and meta-analysis reported that slow acetylators have a significantly higher risk of developing DIH, with an odds ratio of 2.52 (95% CI: 1.95–3.27) (7). In contrast, rapid acetylators may be predisposed to subtherapeutic isoniazid concentrations, which could increase the risk of treatment failure. Consequently, stratified isoniazid dosing according to NAT2 genotype may represent a potential strategy to optimize both safety and therapeutic efficacy.

NAT2 genotype distribution varies markedly by ethnicity. While slow acetylators predominate among Caucasians and Africans (58–60%), Asian populations, including Thais, exhibit lower proportions of slow acetylators (6). Thai studies have reported diverse genotype distributions: slow, intermediate, and rapid acetylators at 37.3, 49.6, and 13.1%, respectively (8). These findings suggest that genotype-guided isoniazid dosing could optimize efficacy and minimize toxicity.

To date, limited economic evidence supports pharmacogenetic-guided TB therapy. A cost-effectiveness study in Brazil, South Africa, and India found NAT2-guided dosing to be cost-effective (9). However, no such analyses have been conducted in Thailand. This study therefore evaluates the cost-utility of NAT2-guided versus standard isoniazid dosing in Thai pulmonary TB patients, providing essential evidence to inform resource allocation and national TB control strategies.

## Materials and methods

### Study design

This study was a model-based economic evaluation that combined a decision tree and a Markov model from a societal perspective to evaluate the costs, outcomes, and utilities of a stratified isoniazid dosing regimen based on NAT2 genotype, compared to the standard regimen, in patients newly diagnosed with pulmonary tuberculosis (PTB). Patients with HIV infection were excluded from the analysis. The NAT2 genotype distribution was categorized into three phenotypes: slow, intermediate, and rapid acetylators.

We projected costs and health outcomes in terms of quality-adjusted life years (QALYs) and compared the two strategies using incremental cost-effectiveness ratios (ICERs). Both costs and outcomes were discounted at an annual rate of 3%.

### Populations

The model simulated cohorts of newly diagnosed pulmonary tuberculosis (PTB) patients, with a starting age at treatment initiation of 56 years. This age was derived from a large observational study conducted in Thailand using data from the National Tuberculosis Information Program (NTIP) database (10).

### Intervention and comparator

The intervention was isoniazid dosing stratified according to the NAT2 genotype (genotype-guided group). A standard body weight of 50 kg was assumed for all patients. This value was derived from data obtained from the TB clinic at Uttaradit Hospital, Thailand, and represented the mean body weight of newly diagnosed pulmonary tuberculosis patients without HIV infection treated at this center.

The genotype-guided isoniazid dosing regimen was based on a randomized controlled trial conducted by Koizumi et al. (11), which recommended weight-based doses of 7.5 mg/kg, 5 mg/kg, and 2.5 mg/kg for rapid, intermediate, and slow acetylators, respectively. In clinical practice, these weight-based doses were approximated to fixed daily doses of 400 mg, 300 mg, and 150 mg, respectively, to improve feasibility and adherence.

The comparator group received dosing based on the National Tuberculosis Control Programme Guideline, Thailand 2021 (12). Isoniazid was administered at 300 mg/day, in combination with rifampicin 600 mg/day, ethambutol 800 mg/day, and pyrazinamide 1,000 mg/day (standard regimen group).

### Model structure and model assumptions

#### Decision tree

**Figure 1:**
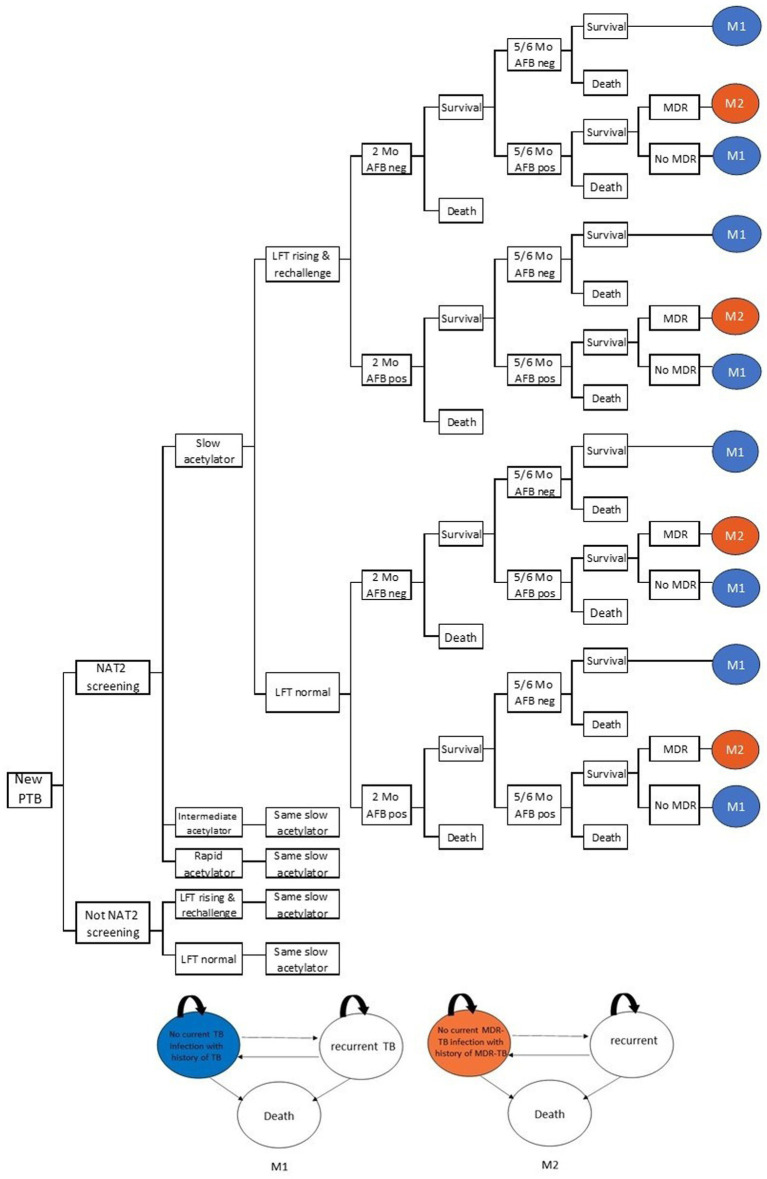
Model structure combined a decision tree and a Markov model. PTB, pulmonary tuberculosis; NAT2, N-acetyltransferase 2 enzyme; MDR TB, multidrug resistance TB; LFT, liver function test; M, month; AFB, acid-fast bacillus; pos, positive; neg, negative.

We assumed that all patients at baseline received a two-month intensive phase consisting of isoniazid, rifampicin, ethambutol, and pyrazinamide, followed by a four-month continuation phase with isoniazid and rifampicin. Individual NAT2 genotypes were linked to key clinical outcomes, including elevated liver enzyme levels, a positive acid-fast bacillus (AFB) smear at two months, treatment failure, mortality, and survival. The time horizon for the decision tree model was set at two years. Patients who developed elevated liver enzymes underwent a one-month rechallenge phase. Final treatment outcomes were evaluated at five to six months (Figure 1).

#### Markov model

Patients who were cured of PTB remained at risk of recurrence and subsequently entered Markov model M1 in the health state “No current TB infection with a history of TB.” From this state, patients could experience recurrent TB or death in the following cycle. Recurrent TB could either transition back to the cured state (“No current TB infection with a history of TB”) or result in death (Figure 1).

Patients who experienced treatment failure could progress to multidrug-resistant tuberculosis (MDR-TB) or remain non-MDR. Individuals who achieved a cure following MDR-TB treatment were assigned to the Markov model state M2 (“No current MDR-TB infection with a history of MDR-TB”) and were at risk of developing recurrent MDR-TB (Figure 1). Similarly, patients with recurrent MDR-TB could either transition back to the cured state (“No current MDR-TB infection with a history of MDR-TB”) or progress to death.

The Markov model was conducted over a lifetime horizon with a cycle length of one year. It was assumed that patients with a history of tuberculosis remain at risk for reinfection with TB, and those with prior MDR-TB may experience reinfection with MDR-TB. This possibility of reinfection represents a key limitation of the present study. The comparator used the same model structure but with different transition probabilities and utilities.

### Input parameters

#### Transitional probabilities

For the NAT2 genotype group, the NAT2 genotype distribution was obtained from the Regional Medical Science Center 2, Phitsanulok, located in northern Thailand (13). Slow acetylators had a higher risk of liver enzyme elevation (6), and those who developed hepatitis had a markedly increased mortality risk (adjusted OR = 12.8) (14). The proportion of AFB smear-positive patients at two months was obtained from the literature (6), with persistent positivity at five months defined as treatment failure. Rapid acetylators showed a higher likelihood of treatment failure than slow or intermediate acetylators (adjusted OR = 1.54) (15). Patients experiencing treatment failure were more likely to progress to MDR-TB (16). Recurrent TB was associated with increased mortality (OR = 1.11; 95% CI 0.61–2.04) (17) and reduced cure rates (18), while MDR-TB was strongly linked to elevated mortality (HR = 7.5) (19) and lower cure rates (20).

In the comparator group, 11.5% of patients experienced liver enzyme elevation (21). The proportion achieving AFB smear negativity at two months was obtained from a systematic review and meta-analysis (22). Treatment failure was derived from a retrospective cohort study (23). TB-related mortality rates were derived from systematic reviews and meta-analyses (24). Patients with a history of PTB had an increased risk of recurrence (RR = 1.98) (25) (see Table 1).

**Table 1:** Input parameters

Parameter	Base value	Range	Distribution	Reference
Distribution of acetylators, %				Medical Sciences Center (13)
Slow	38.78		Beta	
Intermediate	46.21		Beta	
Rapid	15.01		Beta	
Median age in pulmonary tuberculosis, years	56			Sanmai et al. (10)
Probabilities				
AFB positive at 2 months with slow	0	Fixed	Fixed	Surarak et al. (6)
AFB positive at 2 months with intermediate	0.2680	N/A	Beta	Surarak et al. (6)
AFB positive at 2 months with rapid	0.1500	N/A	Beta	Surarak et al. (6)
LFT rising with slow	0.4179	0.1493–1.0000	Beta	Surarak et al. (6)
LFT rising with intermediate	0.1471	0–0.3333	Beta	Surarak et al. (6)
LFT rising with rapid	0.1211	0–0.3333	Beta	Surarak et al. (6)
AFB negative at 2 months with first-line Anti-TB drugs	0.8200	0.7800–0.8600	Beta	Calderwood et al. (22)
Treatment failure in slow, intermediate	0.0370	N/A	Beta	Amorim et al. (15)
Treatment failure in patients with rapid	0.0570	0.0211–0.1550	Beta	Amorim et al. (15)
Treatment failure in first-line Anti-TB drugs	0.0090	N/A	Beta	Gatechompol et al. (23)
Probability of MDR-TB in previously treated in Thailand	0.0970	0.0910–0.1000	Beta	Division of Tuberculosis, Department of Disease Control, Ministry of Public Health (16)
Recurrent of previously TB	0.0031	0.0020–0.0046	Beta	Vega et al. (25)
Recurrent of previously MDR-TB	0.0492	N/A	Beta	Park et al. (20)
Death from first-line Anti-TB drugs	0.0961	0.0701–0.1359	Beta	Romanowski et al. (24)
Death from unsuccess first-line Anti-TB drugs	0.8785	N/A	Beta	Sumetvathaniya (30)
Death from Anti-TB induced hepatitis	0.7255	0.1746–0.9997	Beta	Phasuknit (14)
Death from TB with slow	0.0210	0.0033–0.0191	Beta	Kasamatsu et al. (31)
Death from TB with intermediate	0.0290	N/A	Beta	Kasamatsu et al. (31)
Death from TB with rapid	0.0030	0.0043–0.0303	Beta	Kasamatsu et al. (31)
Cured form recurrent TB	0.6329	N/A	Beta	Hunchangsith et al. (18)
Cured form recurrent MDR-TB	0.1644	N/A	Beta	Park et al. (20)
Hepatitis from first-line Anti-TB drugs	0.1150	0.1010–0.1297	Beta	Wang et al. (21)
Costs*, THB (USD)***				
NAT2 test	1,630** (50)	N/A	Gamma	Gazette (26)
First-line anti-TB drugs	17,598 (536)	N/A	Gamma	Chawetsan et al. (27)
Slow	28,038 (854)	N/A	Gamma	Primary data, Chawetsan et al. (27)
Intermediate	22,652 (690)	N/A	Gamma	Primary data, Chawetsan et al. (27)
Rapid	20,731 (632)	N/A	Gamma	Primary data, Chawetsan et al. (27)
Anti-TB induced hepatitis in Thailand	3,739 (114)	N/A	Gamma	Rochanathimoke et al. (28)
MDR-TB	156,222 (4,760)	N/A	Gamma	Chawetsan et al. (27)
Utilities				
Intensive phase	0.6075	N/A	Beta	Primary data
Continuation phase	0.8035	N/A	Beta	Primary data
Survival	0.9327	N/A	Beta	Primary data
MDR-TB	0.5100	N/A	Beta	Park et al. (32)
MDR-TB after treated	0.8800	N/A	Beta	Park et al. (32)
Hepatotoxicity	0.6670	0.6170–0.7170	Beta	Sadatsafavi et al. (33)
Healthy past treated for TB/MDR-TB	0.9420	N/A	Beta	Suen et al. (34)
Active recurrent TB	0.8100	0.6460–0.9720	Beta	Jit et al. (35)
Death	0	Fixed	Fixed	Suen et al. (34)

*Convert to consumer price index 2024; **Cost to chart ratio; ***32.82 THB = 1 USD (https://www.bot.or.th/th/statistics/exchange-rate.html, 22 August 2025). Slow, slow acetylator; intermediate, intermediate acetylator; rapid, rapid acetylator; MDR-TB, multidrug-resistant TB; THB, Thai Baht; LFT, liver function test.

#### Costs

The cost of NAT2 genotyping was obtained from the Medical Science Center (26). Direct medication costs included expenses for drugs, laboratory tests, service delivery, directly observed therapy (DOT) interventions, and home visit programs. Direct non-medication costs encompassed food, travel, and income losses of caregivers. Costs for treating patients with first-line anti-TB and MDR-TB were obtained from published Thai studies (27), except for direct medication costs associated with NAT2 genotyping, which were collected from Thongsaenkhan Hospital, a secondary care hospital in Uttaradit Province, northern Thailand. Direct non-medical costs were obtained from published Thai studies (27). Costs for anti-TB drug-induced hepatitis were obtained from published Thai studies (28).

#### Utilities

Utilities for PTB were categorized into three health states: intensive phase, continuation phase, and post-treatment survival. These data were collected from primary sources in eight secondary care hospitals in Uttaradit through face-to-face interviews using the Thai version of the EQ-5D-5L, a tool registered and approved by the EuroQol Group. A total of 82 participants were recruited using cluster random sampling across the eight hospitals, with convenience sampling within each cluster. Utilities for MDR-TB, hepatitis, post-treatment (TB/MDR-TB) survival, and active recurrent TB were obtained from published literature. It was assumed that patients within the same health state shared equivalent utility values.

### Data analysis

#### Base-case analysis

To perform the analysis, the incremental cost-effectiveness ratio (ICER) was calculated using the following equation:


$$\text{ICER}=\frac{\text{Cost}(T)-\text{Cost}(C)}{\text{QALY}(T)-\text{QALY}(C)}$$

Where:

Cost(T) = Costs from the NAT2 genotype group,

Cost(C) = Costs from the standard regimen group,

QALY(T) = Quality-adjusted life years from the NAT2 genotype group,

QALY(C) = Quality-adjusted life years from the standard regimen group.

QALY was calculated as:


QALY=Life years×Utility

ICER was used to determine whether the intervention was cost-effective compared to the threshold of 160,000 THB/QALY (4,875 USD/QALY). All costs and outcomes were discounted at an annual rate of 3%, in accordance with Thai HTA guidelines (29).

#### Sensitivity analyses

A one-way sensitivity analysis was performed to evaluate the effect of individual input parameters on ICERs. Each parameter was varied across its reported confidence interval or, when unavailable, within a ± 10% range (costs within a ± 20% range). For the probabilistic sensitivity analysis (PSA), beta distributions were applied to probabilities and utility values, while gamma distributions were applied to cost parameters. Model parameters were randomly sampled 10,000 times according to their respective distributions. Results were illustrated as a scatter plot on the cost-effectiveness plane. Additionally, the probability of the intervention being cost-effective across different willingness-to-pay (WTP) thresholds was presented using a cost-effectiveness acceptability curve.

## Results

The base-case analysis indicated that the lifetime total costs were 28,538 THB (USD 870) for the NAT2 genotype-guided dosing group and 18,727 THB (USD 570) for the standard regimen group, resulting in an incremental cost of 9,810 THB (USD 299). Across all cost components, the NAT2 genotype-guided group incurred higher costs than the standard regimen group, except for expenditures related to first-line anti-tuberculosis drugs.

The estimated life years were 10.65 for the NAT2 genotype-guided group and 8.24 for the standard regimen group, yielding an ICER of 4,062 THB (USD 124) per life-year gained. Similarly, QALYs were 9.92 for the NAT2 genotype-guided group and 7.66 for the standard regimen group, resulting in an incremental gain of 2.26 QALYs. The corresponding ICER was 4,333 THB (USD 132) per QALY gained (Table 2).

**Table 2:** Base-case results.

Outputs	Stratified isoniazid dosing by NAT2 genotype	Standard regimen	Incremental
Total costs, THB (USD)	28,538 (870)	18,727 (570)	9,810 (299)
NAT-2 test	1,583 (48)	0.00	1,582 (48)
First-line anti-TB drugs	603 (18)	17,548 (535)	−16,945 (516)
Treatment for hepatitis	1,034 (32)	516 (16)	518 (16)
Slow acetylator	10,929 (333)	286 (9)	10,643 (324)
Intermediate acetylator	10,521 (320)	276 (8)	10,246 (312)
Rapid acetylator	3,128 (95)	82 (3)	3,046 (93)
MDR-TB	740 (22)	19 (0.6)	721 (22)
Total life-year	10.65	8.24	2.42
Total QALYs	9.92	7.66	2.26
ICER, THB (USD)/Life year	4,062 (124)		
ICER, THB (USD)/QALY	4,333 (132)		

THB, Thai Baht; MDR-TB, multidrug-resistant TB; QALY, quality-adjusted life year; ICER, incremental cost-effectiveness ratio.

### One-way sensitivity analysis

**Figure 2:**
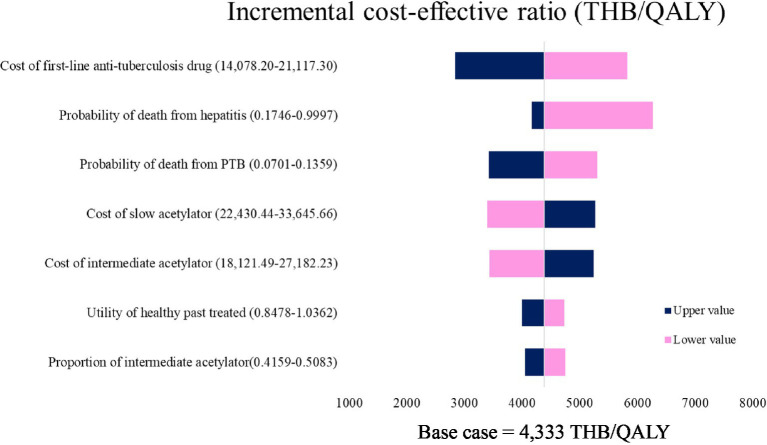
Tornado diagram presenting the results of the one-way sensitivity analysis (stratified isoniazid dosing by NAT2 vs. standard regimen). QALY, quality-adjusted life year; THB, Thai Baht; PTB, pulmonary tuberculosis; LFT, liver function test.

The cost-effectiveness results were robust across all input parameters. However, the cost of first-line anti-TB drugs, the probability of death from hepatitis, the probability of death from PTB, the cost of slow acetylators and the cost of intermediate acetylators exerted the greatest (top five) influence on the ICER values (Figure 2).

### Probabilistic sensitivity analysis

**Figure 3:**
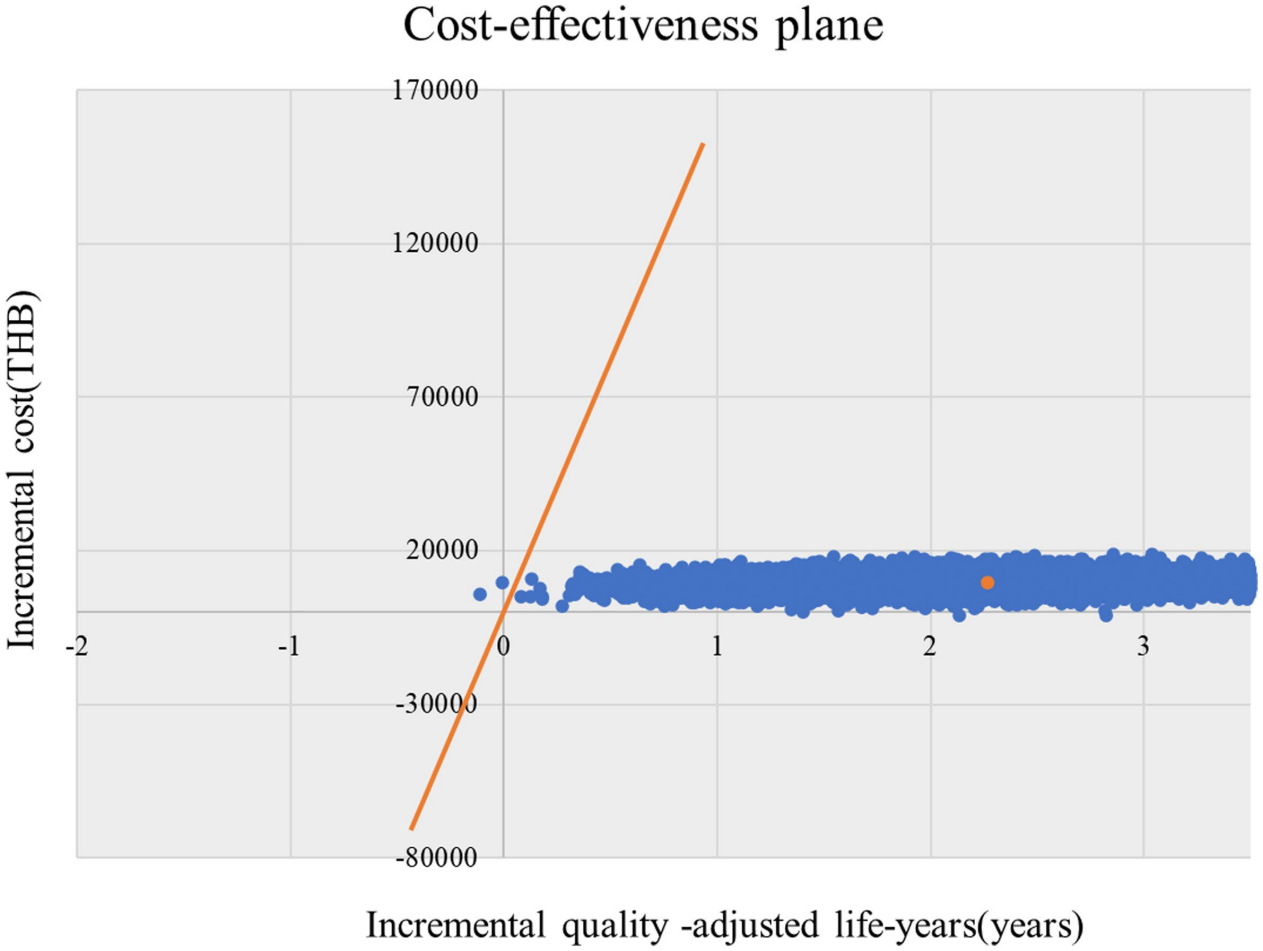
Probabilistic sensitivity analysis (stratified isoniazid dosing by NAT2 vs. standard regimen); cost-effectiveness plane. QALY, Quality-adjusted life year; THB, Thai Baht.

**Figure 4:**
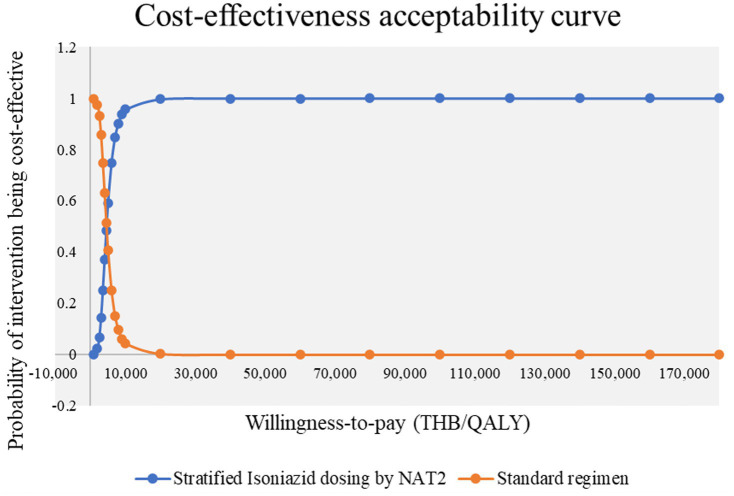
Probabilistic sensitivity analysis (stratified isoniazid dosing by NAT2 vs. standard regimen); cost-effective acceptability curve. QALY, quality-adjusted life year; THB, Thai Baht.

In 10,000 simulations, the net monetary benefit (NMB) was greater than 0 in 99.90% of cases for patients receiving NAT2 genotype-guided isoniazid dosing. ICERs were consistently below the willingness-to-pay threshold, indicating cost-effectiveness (Figures 3, 4).

## Discussion

This study demonstrated that stratified isoniazid dosing based on the NAT2 genotype yielded an ICER far below Thailand’s willingness-to-pay threshold of 160,000 THB per QALY, indicating that the strategy is cost-effective when compared with the standard dosing regimen. The robustness of the results was confirmed through one-way sensitivity analyses, in which all parameters maintained cost-effectiveness despite variations in input values. The probabilistic sensitivity analysis further reinforced these findings, showing a 99.98% probability that NAT2-guided dosing would be cost-effective. Together, these results support the potential value of incorporating pharmacogenetic testing into routine tuberculosis care in Thailand.

Our findings are aligned with previous international economic evaluations. Rens et al. (9) reported that NAT2 genotype-guided isoniazid dosing was cost-effective in Brazil, South Africa, and India, with ICERs below the per-capita GDP in each country. This consistency across diverse healthcare settings suggests that the intervention has global applicability, particularly in regions with a high burden of tuberculosis and diverse NAT2 genotype distributions.

Although the NAT2-guided strategy was expected to reduce the incidence of DIH, our model demonstrated higher DIH-related treatment costs in the NAT2-guided group compared with the standard regimen. This finding may be explained by the relatively high probability of liver enzyme elevation among slow acetylators, resulting in increased costs associated with DIH management despite the overall reduction in hepatotoxicity risk. Nevertheless, the NAT2-guided group incurred higher total costs but achieved greater QALYs gained. This improvement in QALYs likely reflects the reduced quality-of-life impairment typically experienced by patients who develop drug-induced hepatitis.

The strengths of this study include the use of cost data obtained from published literature in Thailand and the incorporation of expenses associated with directly observed therapy and home visits in the model. Despite these strengths, several limitations should be acknowledged. First, the cost of NAT2 genotyping was estimated using data from a secondary-care hospital, which may not capture cost variation across different levels of healthcare facilities or private-sector laboratories. Second, the model assumed fixed transition probabilities between PTB and MDR-TB, although real-world evidence indicates that patients may experience reinfection or follow alternative disease progression pathways. Third, the starting age of 56 years was relatively high and may influence the risk of drug-induced hepatotoxicity and mortality.

The variation in genotype distributions across regions worldwide and differences in the proportion of slow, intermediate, and fast acetylators could meaningfully affect the magnitude of cost savings and clinical benefits. Future research should therefore include region-specific genotype distributions and real-world outcome data from diverse patient populations. Additionally, implementation research is needed to identify barriers to incorporating pharmacogenetic testing into routine TB programs, including laboratory capacity, clinician training, and patient acceptability.

In the long term, integrating pharmacogenetic-guided dosing strategies could contribute to personalized tuberculosis therapy, which aligns with global trends toward precision medicine. As genotyping technology becomes more affordable and accessible, NAT2-guided isoniazid dosing has the potential to reduce the public health burden associated with DIH, improve treatment adherence, and ultimately enhance TB control efforts.

## Conclusion

Stratified isoniazid dosing based on NAT2 genotype was shown to be cost-effective among patients with newly diagnosed pulmonary tuberculosis. However, treatment costs related to anti-tuberculosis drug-induced hepatitis were higher in the NAT2-guided group compared with the standard regimen.

## Data Availability

The raw data supporting the conclusions of this article will be made available by the authors, without undue reservation.
